# Dual roles of mTOR in skeletal muscle adaptation: coordinating hypertrophic and mitochondrial biogenesis pathways for exercise-induced chronic disease management

**DOI:** 10.3389/fmed.2025.1635219

**Published:** 2025-08-29

**Authors:** Yong-Cai Zhao

**Affiliations:** Tianjin Key Laboratory of Exercise Physiology and Sports Medicine, College of Exercise and Health, Tianjin University of Sport, Tianjin, China

**Keywords:** skeletal muscle, mitochondrial biogenesis, hypertrophy, exercise, mammalian target of rapamycin (mTOR)

## 1 Introduction

Resistance training causes muscle hypertrophic remodeling, primarily through mammalian target of rapamycin (mTOR)-mediated protein synthesis (1). Endurance training upregulates adenosine monophosphate-activated protein kinase (AMPK) and peroxisome proliferator-activated receptor γ coactivator 1α (PGC-1α), enhancing mitochondrial biogenesis in skeletal muscle (2). Previous *in vitro* studies proposed antagonistic crosstalk between mTOR and PGC-1α pathways, wherein their signaling axes compete for transcriptional/translational resources in certain cell types (3). For example, AMPK inhibits mTOR, relieving its suppression of mitophagy in skeletal muscle (4). However, a study found that resistance training increased skeletal muscle PGC-1α and mTOR activities in healthy young men, suggesting that the interfering effects might not exist *in vivo* (5). Moreover, a research has demonstrated that mTOR not only exerts its traditional function of driving muscle hypertrophy, but also indirectly promotes muscle PGC-1α signaling in mice (6). Thus, integrative adaptations of skeletal muscle mediated by mTOR are not just confined to controlling muscular growth. mTOR also influences mitochondrial biogenesis signaling. Drugs or exercises targeting the mTOR may yield treatment strategies for the chronic diseases such as diabetes and sarcopenia. Present article mainly discussed the regulation of mitochondrial biogenesis by mTOR in skeletal muscle.

## 2 The classical role of mTOR in regulating skeletal muscle hypertrophy with mechanical loads

mTOR positively regulates protein synthesis and ribosomes biogenesis, contributing to the skeletal muscle hypertrophic response (7). The skeletal muscle hypertrophic adaptation is meditated by both mTOR complex 1 (mTORC1) and mTOR complex 2 (mTORC2). mTORC1 is the primary kinase responsible for controlling muscle hypertrophy with mechanical loads (7). For example, genetic deleting tuberous sclerosis complex (TSC, a mTORC1 inhibitor) promoted the mTORC1 signaling, and hypertrophic adaptation and atrophy-resistance of skeletal muscle also occurred after denervation operation in mice (8). The muscle hypertrophic response is mediated by the regulatory-associated protein of mTOR (Raptor) which is an essential part of mTORC1. After mechanical overload, muscle Raptor-deletion mice exhibited blunted mTORC1 signaling and attenuated muscle mass accrual (8). Muscle hypertrophy and protein synthesis were accelerated when mTORC1 was activated by the upstreaming regulator protein kinase B (Akt), whereas rapamycin-inhibited mTORC1 blunted mechanical overload-induced hypertrophy in mice (9, 10).

After activation by resistance exercise or/and amino acid ingestion, mTORC1 can localize with lysosomes and move toward the cell membrane, enhancing protein translation and accretion in skeletal muscle (11). Resistance exercise acutely increased mTORC1 activity and mTOR-lysosome translocation of II fibers 3 h post-exercise in young men (12). Exercise combined with feeding may result in synergistic effect because resistance training plus protein-carbohydrate feeding increased muscle mTORC1 signaling greater than exercise alone in young men (13). Eight weeks of resistance training further enhanced the Akt/mTORC1 phosphorylation in skeletal muscle, driving muscle hypertrophy in men (14). Therefore, mTORC1 is an essential regulator of the muscle hypertrophic adaptation by resistance training. A study found mTORC2 also positively regulates the muscle hypertrophic change with muscle contraction because the reduced extent of protein synthesis and hypertrophic response under the inhibition of both mTORC1 and mTORC2 was higher than that under the single inhibition of mTORC1 (15). However, the precise mechanism of mTORC2-mediated muscle hypertrophy with muscle contraction is unclear.

A key downstream protein of mTORC1 is ribosomal S6 protein kinase 1 (S6K1), which promotes the functions of eukaryotic translation initiation factor 4B, eukaryotic elongation factor 2, and ribosomal protein S6, accelerating protein synthesis and the hypertrophic alteration in skeletal muscle (7). Either high load resistance training or low load resistance training with more fatigue, increased S6K1 phosphorylation and mTORC1-associated signals of skeletal muscle in humans during recovery period, which meant S6K1 is implicated in the skeletal muscle hypertrophic response to mechanical overload (16). Eukaryotic initiation factor 4E binding proteins (4E-BPs) are another downstream target of mTORC1. Once phosphorylated by mTORC1, 4E-BPs dissociate from eukaryotic translation initiation factor 4E (eIF4E), enabling eIF4F formation, which then increases the ribosomal biogenesis contributing to the skeletal muscle hypertrophy (7).

Upstream regulators of mTORC1 involved in muscle hypertrophic remodeling include insulin-like growth factor-1 (IGF-1), extracellular signal regulated kinase (ERK), peroxisome proliferator-activated receptor γ coactivator 1α4 (PGC-1α4), and diacylglycerol kinase ζ (DGKζ) (7). These regulators modulate mTORC1 in an independent manner. Most growth factors that stimulate mTORC1 are blocked by TSC. IGF-1 is a classic regulator for protein synthesis in skeletal muscle. Following feeding or mechanical load stresses, IGF-1 binds to IGF-1 receptor and phosphorylates an intracellular adaptor protein insulin receptor substrate-1, which phosphorylates phosphoinositide 3-kinase (PI3K) followed by Akt activation. Akt inhibits tuberous TSC, resulting in activation of small G protein Ras homolog enriched in brain (Rheb), which then activates mTORC1 in skeletal muscle (17). ERK also inhibits TSC to initiate the skeletal muscle mTORC1 activation in the beginning of mechanical overload stresses independently of IGF-1 signaling (18). DGKζ converts diacylglycerol into phosphatidic acid, which binds to the FKBP12-rapamycin binding domain of mTORC1 and enhances mTORC1 activity, which is critical for the skeletal muscle hypertrophic response to mechanical overloads (19). PGC-1α4 differs from other family members of PGC-1α in that resistance training preferentially initiates PGC-1α4 transcriptional expression, which then leads to mTORC1-mediated muscle hypertrophic signaling (20).

## 3 The potential role of mTOR in regulating mitochondrial biogenesis of skeletal muscle

### 3.1 mTOR does not affect muscle PGC-1α-mitochondrial biogenesis signals *in vivo*

PGC-1α regulates skeletal muscle mitochondrial biogenesis with exercise. Conventional view holds that PGC-1α-driven mitochondrial biogenesis competes with mTORC1-mediated protein synthesis, but this opinion has been challenged for that resistance training simultaneously elevates both pathways, suggesting their synergistic potential in muscle adaptation in humans (2). Animal study also reported that AMPK and Raptor activities of skeletal muscle were enhanced following resistance training in rats, suggesting that mTOR and PGC-1α signals can be elevated simultaneously *in vivo* (21). Additionally, emerging evidence has demonstrated that mTOR may be involved in maintaining or enhancing PGC-1α signaling. In humans, two studies have found that resistance training combined with endurance training could amplify the muscle mitochondrial biogenesis signaling, particularly enhancing the muscle mitochondrial state 3 respiration more efficiently in the elderly (22, 23).

### 3.2 Evidence of the regulation of mitochondrial biogenesis by mTORC1

The interaction between mTORC1 and PGC-1α may be related to their shared target protein: Yin-yang1 (YY1), because experiment demonstrated mTORC1 phosphorylates and activates the YY1, which then promotes the PGC-1α-mediated mitochondrial biogenesis (24). Mice with muscle-specific deletion of YY1 showed the reduced levels of muscle mitochondrial content and oxidative phosphorylation (25). Another investigation also demonstrated that Raptor of mTORC1 is required in promoting skeletal muscle mitochondrial biogenesis upon Akt activation, because muscle-specific deletion of Raptor in mice impairs mTOR signaling, reducing both hypertrophy and mitochondrial protein content (6).

A recent study demonstrated that 4-week of L-arginine supplementation improved exercise performance, mitochondrial transcription factor A (Tfam), and PGC-1α genes in the gastrocnemius of mice, but these adaptations and mTOR phosphorylation activity were abolished by the mTORC1 inhibitor rapamycin (26). Additionally, supplementation of polygonatum sibiricum polysaccharide in the aged mice enhanced muscle mass; this nutrient also reduced the level of reactive oxygen species (ROS), benefiting mitochondrial biogenesis in senescent C2C12 cells; similarly, inhibition of mTORC1 by LY294002 also decreased mitochondrial membrane potential and led to excessive production of ROS *in vitro* (27). Ashwagandha extract supplementation in aged mice increased skeletal muscle mass, coinciding with elevated mTOR activity and PGC-1α expression; however, the direct causal relationship requires further validation due to the multi-compound nature of plant extracts (28). Above studies totally suggested that mTORC1 may be required for the nutrients-induced PGC-1α signaling of mitochondrial biogenesis in skeletal muscle. It is noted that long-term and hyperactivated mTORC1 (e.g., in TSC1 knockout model) exacerbates mitochondrial dysfunction in muscle (29). In the skeletal muscles of older adults with sarcopenia, mTORC1 may exhibit hyperactivated and reduce protein synthesis, impair mitophagy, and disrupt mitochondrial biogenesis (30), suggesting that intermittent activation of mTOR (e.g., exercise stimulation) rather than overactivation positively regulates mitochondrial biogenesis in skeletal muscle. Thus, resistance training combined with endurance training may be suitable for older adults because endurance training-induced AMPK activation inhibits the hyperactivation of mTOR, benefiting the mitochondrial biogenesis besides treatment of sarcopenia. In MEFs cells, mTORC1 activation increased mitochondrial state 3 respiration and ATP turnover; in MCF 7 cells, the loss of 4E-BPs attenuates the transcription of mTORC1-induced mitochondrial biogenesis genes, including Tfam and ATP5O (ATP synthase O subunit) (31). While *in vitro* findings from MEFs and MCF7 cells provide mechanistic insights, the extrapolation to skeletal muscle physiology requires caution due to tissue-specific regulatory networks governing mitochondrial biogenesis.

**Figure 1:**
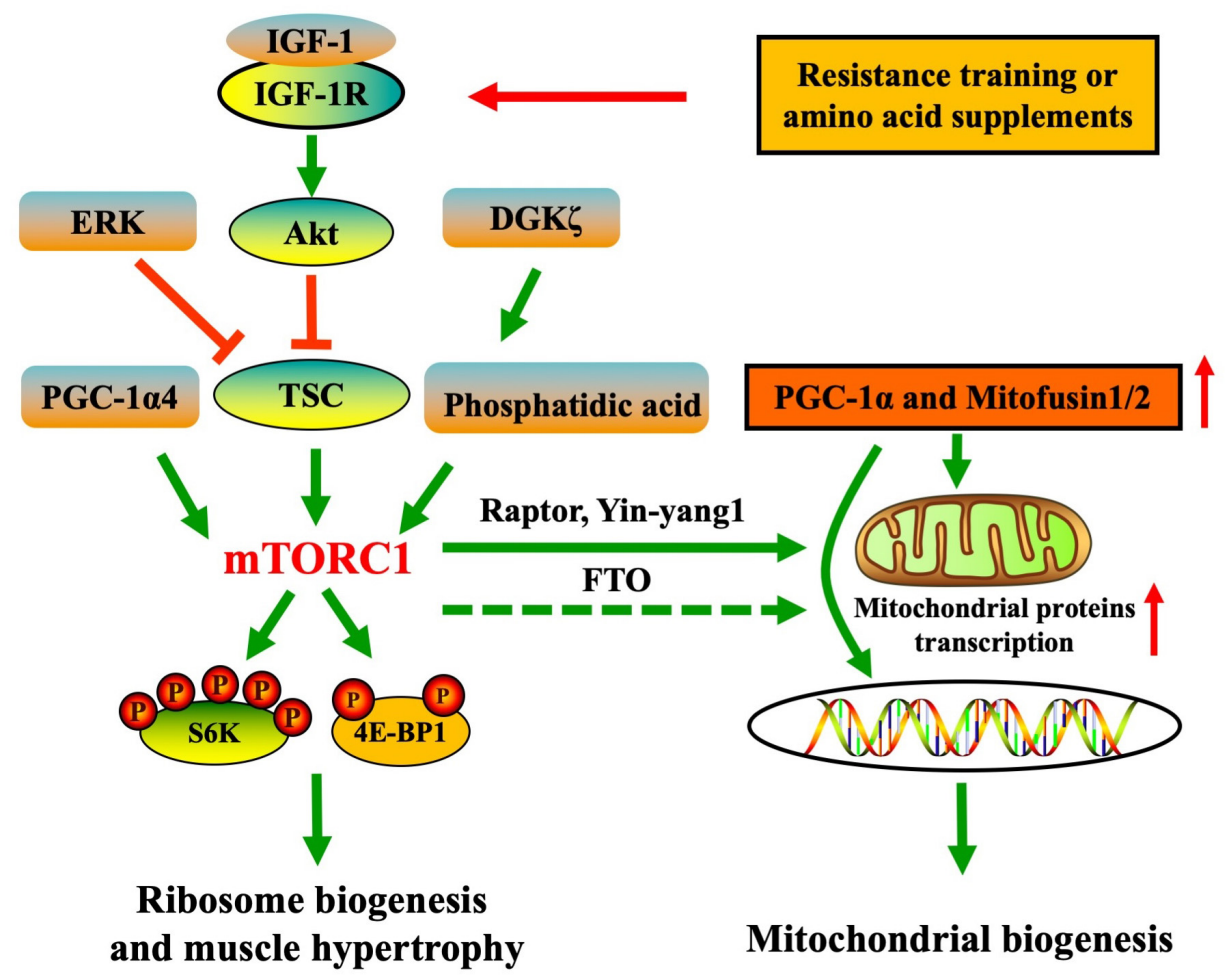
Resistance training and nutritional supplementation elicit muscle mTOR-involved hypertrophic signaling and potentially contribute to PGC-1α signaling of mitochondrial biogenesis.

Therefore, current studies initially demonstrated that mTOR regulates the skeletal muscle PGC-1α pathway, especially being implicated in the nutrients-induced mitochondrial biogenesis (Figure 1). By contrast, chronic hyperactivation of mTOR induced by aging inhibits mitochondrial biogenesis.

### 3.3 Evidence of the regulation of mitochondrial biogenesis-related function by mTORC1

mTOR also regulates muscle mitochondrial dynamics (mitochondrial fusion and fission) (Figure 1). 14-day functional overload (soleus/gastrocnemius removal) in mice induced plantaris hypertrophy, coinciding with elevated mitofusin 2 and optic atrophy 1 proteins contents (enhanced mitochondrial fusion). These mitochondrial adaptations and protein synthesis improvement were inhibited by rapamycin administration, suggesting that mTORC1 might be responsible for enhancing the mitochondrial fusion response in the skeletal muscle (10). The same group also found calorie restriction induced mitochondrial fragmentation of skeletal muscle in mice, which was mediated by dynamin-related protein 1, but this process was partially suppressed by mTORC1 inhibition (32). Mitochondrial fusion and fission participate in the mitochondrial biogenesis process by importing the new components for mitochondrial network in muscle cells (33). Thus, it can be inferred that mTORC1 may regulate mitochondrial biogenesis through controlling the mitochondrial dynamics. During myoblasts differentiation model, Akt/mTOR pathway activation increased the number of mitochondrial DNA (mtDNA) copies and enhanced mitochondrial biogenesis, whereas inhibition of IGF-1 reducing mTOR signals increased the mitochondrial ROS and resulted in a high level of mitochondrial apoptosis signaling (34). Moreover, it was found that fat mass- and obesity-associated (FTO) gene is essential for maintaining the mitochondria biogenesis, ATP content, and mitochondrial DNA copy during skeletal muscle differentiation in mice. Rapamycin blocking muscle mTORC1 suppressed the FTO-induced PGC-1α transcription and affected the muscle differentiation (35), which indicated that mTOR is involved in the muscle differentiation through mitochondrial biogenesis pathway but the link between PGC-1α and mTOR is unclear.

Above studies suggested that mTOR or mTOR upstream signals positively affect the mitochondrial dynamics, mtDNA synthesis, and mitochondrial ROS in skeletal muscle, which may benefit the PGC-1α signaling for mitochondrial biogenesis. It is necessary to verify whether mitochondrial dynamics proteins and FTO act any roles between mTOR and PGC-1α in skeletal muscle.

## 4 Discussion

In skeletal muscle, emerging evidence demonstrated the competition between mTOR and PGC-1α signals may not exist *in vivo*. In nutritional supplement or exercises, mTOR may control the PGC-1α pathway via YY1, 4E-BPs, Raptor, and other unknown ways. mTOR coordinates mitochondrial dynamics (fusion/fission balance) and ROS homeostasis, potentially modulating mitochondrial biogenesis.

In future, it is urgent to study the interaction between muscle mTOR and PGC-1α, as well as how mTOR influences PGC-1α in exercise and nutrients interventions. mTOR should not be considered as a kinase that is only for cell growth. In resistance training condition, activation of mTOR pathway needs the coordination with PGC-1α-meditated mitochondrial biogenesis because protein synthesis requires more ATP supply. Thus, investigation on the role of mTOR in muscle mitochondrial biogenesis is essential for adjusting exercise and nutritional methods to maximize aerobic capacity for sarcopenia patients. For instance, we can construct combined resistance and endurance trainings. The resistance training session may benefit the non-mTOR protein synthesis pathways and the endurance training session restores the normal function of mTOR by AMPK's inhibitory effect. A meta-analysis demonstrated that resistance combined with endurance exercise elicited significant improvements in sarcopenia-related parameters (36). However, current researches on exercise interventions for sarcopenia still lacks investigation into skeletal muscle mTOR signaling. Future studies are anticipated to validate whether multicomponent exercise can more effectively modulate mTOR signaling dysfunction in animal models. mTOR exerts function in mediating glucose metabolism and insulin signaling. Investigation the effect of mTOR on the mitochondrial function in muscle can support the strategies for understanding of the mechanism of exercise treatment on the diabetes and other chronic diseases.
